# The Immune Landscape of Chondrosarcoma: A Review of Tumor-Related Immune Cells

**DOI:** 10.3390/cancers18172889

**Published:** 2026-09-07

**Authors:** Maria T. Silvaggio, Christian P. Tabler, Sila Basbay, Kurt Weiss, Karen Schoedel, Ines Lohse

**Affiliations:** 1 Department of Orthopedic Surgery, University of Pittsburgh School of Medicine, Pittsburgh, PA 15219, USAweiskr@upmc.edu (K.W.); 2 Orland Bethel Musculoskeletal Research Center Summer Program, University of Pittsburgh, Pittsburgh, PA 15261, USA; 3 UPMC Hillman Cancer Center Academy, University of Pittsburgh, Pittsburgh, PA 15213, USA; 4 Orland Bethel Musculoskeletal Research Center, Department of Orthopedic Surgery, University of Pittsburgh, Pittsburgh, PA 15213, USA; 5 Hillman Cancer Center, University of Pittsburgh, Pittsburgh, PA 15232, USA; 6 Department of Pathology, University of Pittsburgh School of Medicine, Pittsburgh, PA 15261, USA

**Keywords:** chondrosarcoma, tumor immune microenvironment, macrophage, T-cells, B-cells, natural killer, neutrophil, cancer, expression

## Abstract

Chondrosarcoma (CS) is a rare cancer comprising a heterogeneous group of malignant cartilage-forming tumors. CS is considered a “surgical disease” characterized by high levels of resistance to chemotherapy, radiation therapy, and limited response to immune checkpoint inhibitors. Due to the rarity of the disease and lack of research models, the impact of the CS microenvironment on tumor development and progression remains elusive. Nevertheless, a few recent studies have begun to interpret the composition and function of the CS tumor immune microenvironment (TME), elucidating its contribution to immune evasion and tumor progression. This review summarizes current findings on the immune cell landscape of CS, highlighting key immune cells, their characteristics, and their potential as therapeutic targets.

## Simple Summary

Chondrosarcoma is a rare type of bone cancer that is difficult to treat, especially when it has spread or returned. Surgery remains the main form of treatment. There are limited options for patients with advanced disease, so researchers are increasingly studying the tumor microenvironment, which contains many different types of cells, including various immune cells. Immune cells such as macrophages, T-cells, B-cells, natural killer cells, and neutrophils may influence how chondrosarcoma grows, spreads, and responds to treatment. However, much remains unknown about how these cells directly affect chondrosarcoma. Many existing studies include small numbers of patients, use laboratory or animal models, or only show an association rather than a direct cause. More research is needed to understand these interactions, identify markers for predicting outcomes, and determine whether treatments that target the immune system could benefit patients. Larger patient studies and improved research methods will be important for future progress.

## 1. Introduction

Chondrosarcoma (CS) accounts for about 20% of primary bone tumors and is most prevalent in adults. Conventional CS (C CS) makes up 80–85% of cases and is graded based on morphology and cellularity. Low-grade tumors (Grade I) are characterized by high survival rates, with a 10-year survival rate of about 85%, while high-grade tumors (Grade III) display poor outcomes, with 19–29% survival [1,2,3]. Dedifferentiated CS (DD CS), which comprises around 10% of cases, displays an aggressive phenotype and features a mix of low-grade cartilage-matrix-producing tumor and high-grade sarcoma tissue without cartilage matrix. DD CS is characterized by a higher risk of early metastasis and a poorer survival rate. When considering cancers of the bone and soft tissue, the gold standard of treatment is complete surgical resection of the tumor, and this remains the most effective treatment for CS, especially in early-stage disease [3]. Less invasive therapies along with better predictors of diagnosis and prognosis are becoming increasingly sought after.

**Figure 1:**
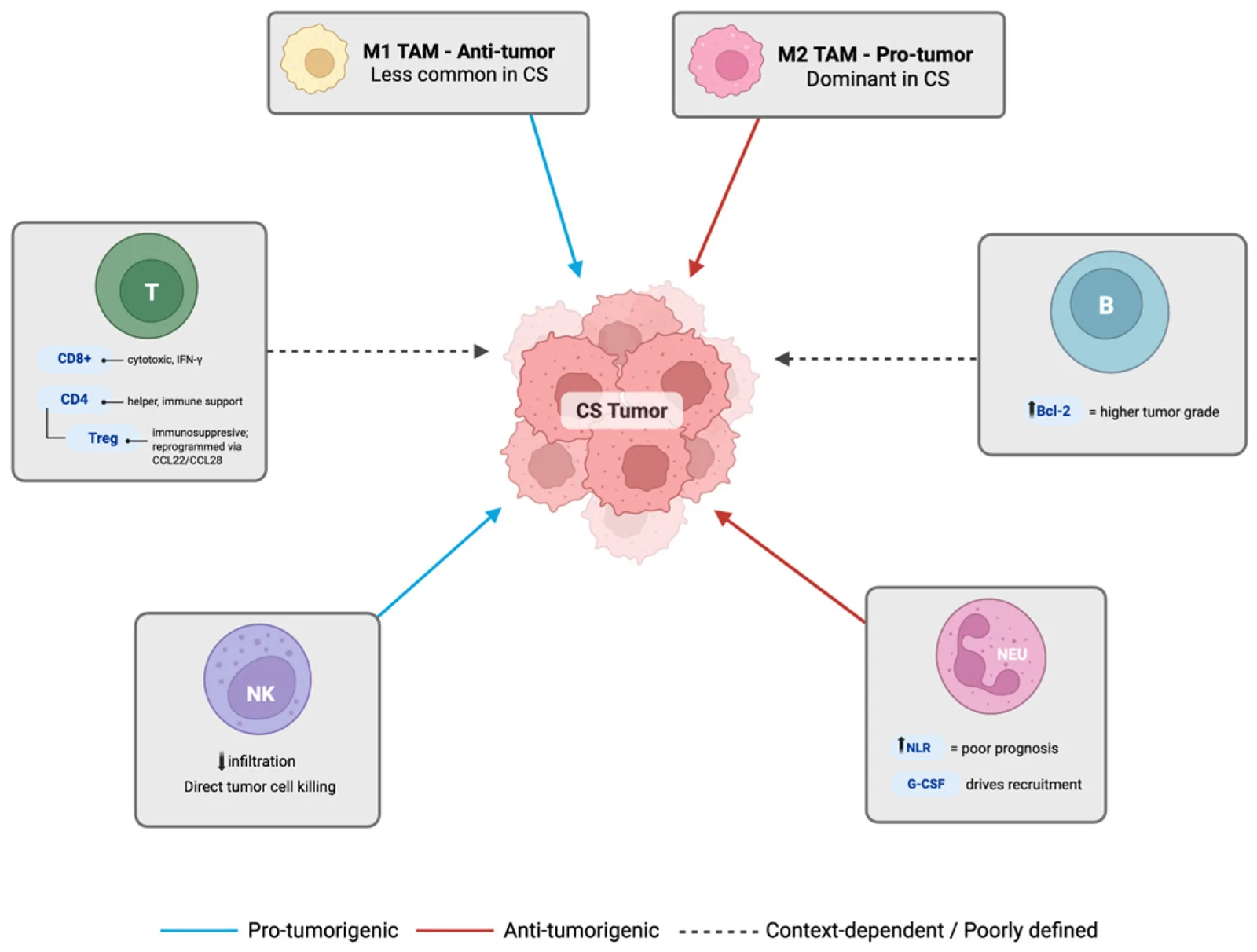
Immune-cell landscape of the chondrosarcoma tumor microenvironment. Major immune populations, including TAMs, T-cells, B-cells, NK cells, and neutrophils, are shown with their reported associations with tumor progression, immune activity, and prognosis. Solid arrows indicate pro- or anti-tumorigenic effects, while dashed arrows indicate context-dependent or poorly defined relationships. Abbreviations: CS, chondrosarcoma; TAM, tumor-associated macrophage; Treg, regulatory T-cell; NK, natural killer; NLR, neutrophil-to-lymphocyte ratio; G-CSF, granulocyte colony-stimulating factor; Bcl-2, B-cell lymphoma 2.

The CS tumor microenvironment (TME) is a complex and organized ecosystem comprising malignant cells with an array of non-malignant cells (Figure 1). Some of these cell types include immune cells, endothelial cells and pericytes, embedded in an extracellular matrix of collagen and laminin in DD CS tumors, while grade 1, 2 and 3 tumors contain malignant chondrocytes embedded in lacunae in cartilage matrix. The foci of vascularized stroma associated with immune cells are present between tumor lobules. The CS TME remains poorly characterized but is increasingly recognized as a critical driver of tumor progression and therapeutic resistance, although the roles of most immune populations, including B-cells and cytotoxic T-cells, have yet to be clearly defined [4]. This is not for lack of effort, as multiple groups are actively undertaking TME research in CS. However, due to low incidence rates and the scarcity of patient specimens for research purposes, progress has remained steady but slow.

## 2. Literature Search Methodology

**Figure 2:**
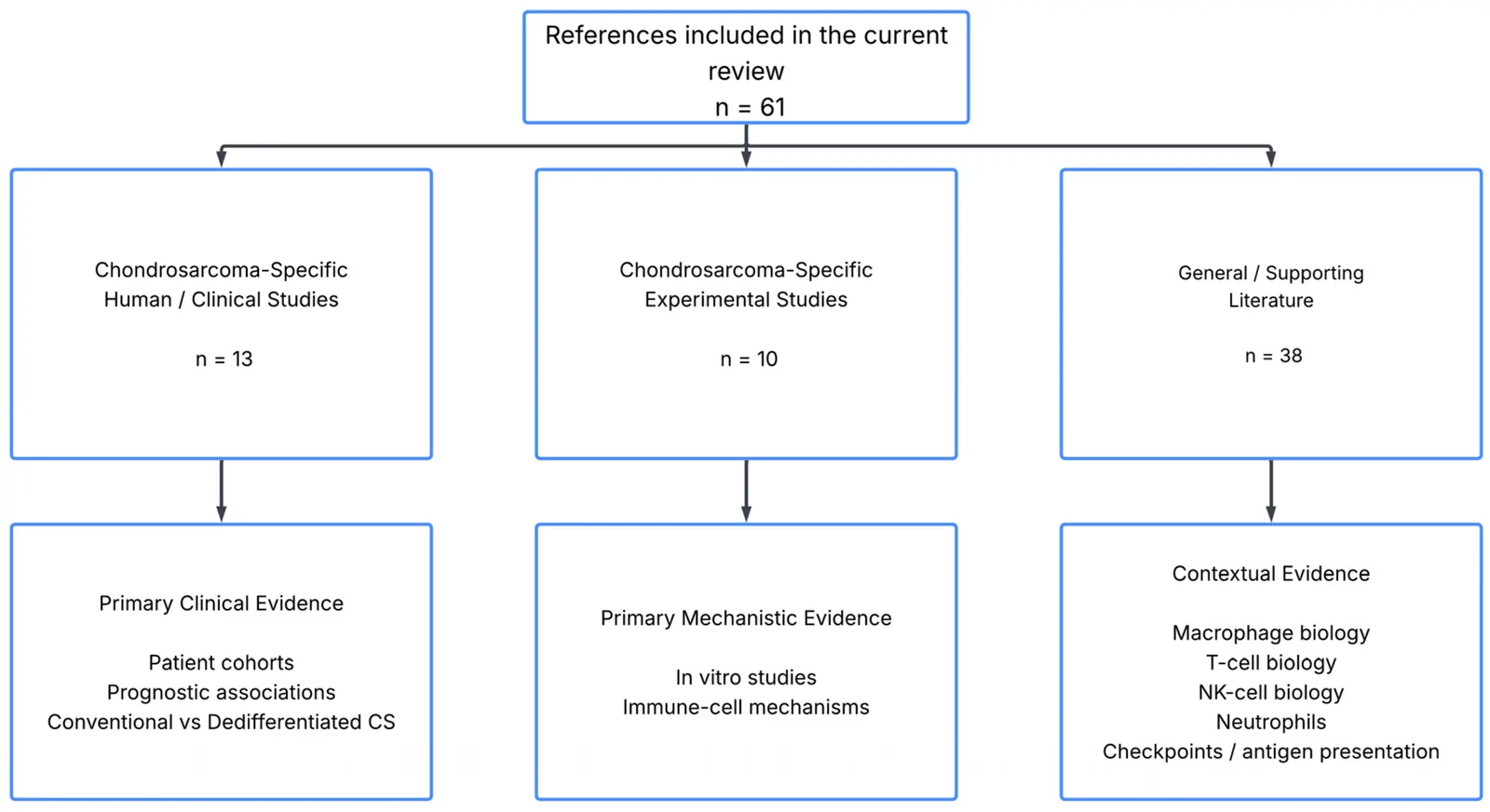
Chondrosarcoma literature evidence framework. Organization of the 62 references included in the current review according to their primary evidentiary role. References were categorized as chondrosarcoma-specific human/clinical studies (n = 14), chondrosarcoma-specific experimental studies (n = 10), or the general/supporting literature (n = 38). Clinical studies primarily provided evidence from patient cohorts, prognostic associations, and comparisons between conventional and dedifferentiated chondrosarcoma. Experimental studies provided primary mechanistic evidence, including in vitro investigations of immune-cell mechanisms in chondrosarcoma. The general/supporting literature provided contextual evidence regarding macrophage, T-cell, natural killer (NK)-cell, and neutrophil biology, as well as immune checkpoints and antigen presentation, where chondrosarcoma-specific evidence remains limited [1,2,3,4,5,6,7,8,9,10,11,12,13,14,15,16,17,18,19,20,21,22,23,24,25,26,27,28,29,30,31,32,33,34,35,36,37,38,39,40,41,42,43,44,45,46,47,48,49,50,51,52,53,54,55,56,57,58,59,60,61].

With the scope of published research on the TME and its role in CS being limited, the literature search strategy was intentionally broad. Only primary research articles were selected. In searching for sources, key terms such as CS and TME were inputted into various online search engines, with each resulting source being read and considered for inclusion. To be eligible for inclusion in this review, the source must have been published after 1998. As outlined in Figure 2, each source was sorted into one of three categories: CS clinical studies, CS experimental studies, and the general supporting literature. Table 1 supplements Figure 2 through the summarization of each clinical and experimental study used.

**Table 1:** Summary of Included Clinical and Experimental Studies. Studies are summarized by study type, population/model, immune/TME component, and main findings.

Reference	Study Type	Population/Model	Immune/TME Component	Main Finding Relevant to Review
[1]	Human Clinical	Patients with chondrosarcoma	General CS prognosis	Evaluated clinical outcomes in bone chondrosarcoma.
[2]	Human Clinical/database	National Cancer Data Base	General CS prognosis	Provides large-scale clinical/outcome information for chondrosarcoma.
[3]	Human CS study	27 conventional CS + 29 dedifferentiated CS samples	TAMs, CSF1R	DD CS showed greater CSF1R+ macrophage infiltration; CSF1R expression was associated with metastatic progression.
[8]	Human CS study	26 conventional CS samples	CD163 + TAMs	Greater CD163+ macrophage infiltration was associated with tumor severity; macrophages were prominent in the CS TME.
[13]	Experimental CS	CS models examining monocyte recruitment	Monocytes/TAMs	Investigated inhibition of monocyte recruitment as a strategy to reduce pro-tumor TAM activity.
[17]	Experimental CS	Human monocytes + CH2879/JJ012 CS cells; 3D spheroid model	TAMs	Developed a 3D CS co-culture model for studying TAM-targeted therapies.
[25]	Human CS study	48 conventional CS + 24 DD CS; immune profiling	TILs, CD4/CD8, HLA, checkpoints	Higher immune infiltration/checkpoint expression was observed in aggressive CS, with important differences between conventional high-grade and DD CS.
[27]	Human CS study	Fresh conventional CS tissue	Multiple immune populations	Multi-omics profiling identified immune subgroups and potential immunotherapy candidates.
[28]	Human CS study	Dedifferentiated CS	PD-L1, CD4, CD8, CD20, PU.I	IHC identified immune-cell/checkpoint markers; CD20+ B-cell infiltration was associated with disease stage.
[29]	Experimental CS	Swarm rat chondrosarcoma model	BCL-2 family/apoptosis	ABT-737 targeting of BCL-xL reduced tumor growth.
[40]	Human clinical	23 DD CS patients	Peripheral NLR	Higher preoperative NLR was associated with poorer prognosis.
[41]	Human case report	72-year-old male with DD CS	Neutrophils/G-CSF	Leukocytosis, neutrophilia, and elevated G-CSF were reported in DD CS.
[42]	Human clinical	81 patients with conventional CS	Peripheral NLR	Elevated pretreatment NLR was associated with poorer prognosis.
[46]	Human CS study	137 CS patients, various subtypes	PD-L1, T cells, HLA	PD-L1 expression was reported specifically in DD CS; a subset showed increased T-cell infiltration associated with PD-L1 expression.
[48]	Experimental/CS tissue + treatment study	207 CS samples; experimental treatment models	Survivin/apoptosis	Survivin was overexpressed in CS and investigated as a potential therapeutic target.
[51]	Human CS/experimental biology	Human chondrosarcoma	VEGF/angiogenesis	Demonstrated VEGF secretion by human CS and its relationship to tumor angiogenesis.
[52]	Experimental CS	Human CS cells	CCl5/VEGF/angiogenesis	CCL5 promoted VEGF expression and angiogenesis in human CS cells.
[53]	Experimental CS	Human CS cells	Adiponectin/VEGF	Adiponectin promoted VEGF-A-dependent angiogenesis through PI3K/Akt/mTOR/HIF signaling.
[54]	Experimental CS	Human CS cells	Amphiregulin/VEGF	Amphiregulin increased VEGF-A production and promoted angiogenesis.
[55]	Experimental CS	Human CS cells	Resistin/VEGF	Resistin promoted VEGF-A-dependent angiogenesis through miRNA regulation.
[56]	Experimental CS	CS cell lines + mouse xenograft model	CSCR4/SDF1, hypoxia, invasion	CXCR4/SDF1 signaling increased invasion; CXCR4 inhibition reduced VEGF, tumor growth, and lung metastases in mice.
[58]	Experimental CS	CS cells/chondrocytes under hypoxia	HIF-1α/VEGF	Hypoxia increased HIF-1α and VEGF expression, supporting a role for hypoxia in angiogenesis.
[61]	Human Clinical/Observational	230 human sarcoma tumor specimens: 45 chondrosarcomas, 45 liposarcomas, and 140 undifferentiated pleomorphic sarcomas.	PD-L1 expression; immune checkpoint signaling	PD-L1 was expressed in a subset of chondrosarcomas, supporting the potential relevance of the PD-1/PD-L1 immune checkpoint pathway as a therapeutic target.

## 3. Tumor-Associated Macrophages

Outside of the tumor environment, mature macrophages are classified into two subgroups: classically activated and alternatively activated macrophages [5]. In the presence of factors such as lipopolysaccharide (LPS), interferon (IFN)-γ or tumor necrosis factor (TNF)-α, macrophages adopt a pro-inflammatory phenotype, designated as classically activated [6]. In contrast, alternatively activated macrophages are involved in tissue repair, wound healing, and immune suppression.

In tumors, macrophages are recruited from circulating blood monocytes and differentiated within the tumor microenvironment [7].

In C CS, tumor-associated macrophages (TAMs) are typically sparse and restricted to the area surrounding the periphery of the tumor, creating an overall immune-excluded phenotype. In contrast, DD CS exhibits dense intratumoral TAM infiltration, particularly CD68+ and CD163+ macrophages [3]. Furthermore, from a cohort of 26 C CS samples, a positive correlation between tumor severity and CD163+ infiltration into the tumor from the microenvironment was observed. This suggests the role of the CD163+ macrophage as one that promotes tumor proliferation and development, although functional studies demonstrating a direct role remain limited [8]. Beyond TAM density within the tumor, functionality is determined by polarization within the TME. Tumor-associated macrophages exhibit phenotypic and functional plasticity, and the classically and alternatively activated categories do not fully encompass the heterogeneous states observed in tumors. Under inflammatory stimulation, TAMs can adopt phenotypes characterized by the production of pro-inflammatory cytokines that can promote the killing of tumor cells or elicit vascular endothelial damage [9]. However, available evidence suggests that immunosuppressive TAM phenotypes predominate in CS, and their activation prompts angiogenesis and extracellular matrix remodeling. This subsequently supports greater tumor growth, immune evasion, and metastasis in the sarcoma-associated TME [9].

Another TAM subtype identified within the CS TME is the colony-stimulating factor 1 receptor (CSF-1R+) macrophage. This cell expresses the CSF-1R gene, and when activated by CSF-1, it is involved in processes related to macrophage survival, dedifferentiation, and proliferation [10]. Within a cohort of 27 C CS and 29 DD CS samples, CSF-1R+ macrophage infiltration along the periphery of the tumor was more frequent in DD CS samples (84.6%) than C CS samples (64%), and a positive, statistically significant correlation was identified between CSF-1R+ expression and metastasis progression [3]. The high prevalence of CSF-1R+ macrophage infiltration suggests that their presence may help to establish an immunosuppressive TME.

A third classification of macrophages has been proposed and identified by some researchers. This classification, referred to as M3, represents macrophages with an intermediate or reprogrammed phenotype, which causes them to be both pro- and anti-tumoral depending on context [11]. The M3 classification has not been standardized. Intermediate macrophages, specifically those of FABP4+, have been identified in osteosarcoma by Wu et al. FABP4+ was identified in the primary tumor through scRNA sequencing [12]. However, none of the literature documents the existence of M3 macrophages within CS or its associated TME.

With limited information on the CS TME, few research endeavors centered around macrophages in CS have developed sufficiently to arrive at ideas for therapy. However, some are underway. In the study by Simard et al. [8], the effectiveness of two reagents, mifamurtide and liposome clodronate, was examined. Mifamurtide functions as an anti-cancer drug that promotes macrophage phenotype conversion from alternatively activated to classically activated types, and liposome clodronate functions as a macrophage-depleting reagent. Both reagents yielded a reduction in CD163+ cells and increased animal survival [8]. Yang, L., et al. proposed the idea of TAM reprogramming through pseudomonas aeruginosa mannose-sensitive hemagglutinin. This avenue has the potential to promote macrophage reprogramming toward inflammatory phenotypes capable of enhancing anti-tumor immunity [13,14]. Using human monocytes and the CHS cell lines CH2879 and JJ012, Quoniou et al. developed a 3D spheroid co-culture model for chondrosarcoma. These co-culture models reproduced the characteristics of chondrosarcomas in vitro, supporting the future use of these spheroid models for the development of TAM-targeted therapies [15].

Also studied by Minpoli et al. was macrophage interaction with stromal components within the TAM. Through examination and analysis of CS spheroids and monocytes within a fibroblast collagen matrix, it was demonstrated that tumor-derived signals promoted a tumor-supportive macrophage phenotype, enhancing tumor-cell invasion [13]. Although the specific contributions of fibroblasts to macrophage polarization were not independently evaluated, research of this kind provides the foundations for further study on stromal–immune interactions in the CS TME.

## 4. T-Cells

Common lymphoid progenitors (CLPs) are stem cells from which T-cells differentiate into two distinct subtypes, namely cytotoxic T-cells (CD8+) and helper T-cells (CD4+) [16]. Cytotoxic T-cells serve to kill cells directly, while helper T-cells work to signal and support various immune cell types [17]. In cancer, CD8+ cells detect tumor antigens and, with the secretion of (IFN)-γ, cause tumor cell death and facilitate the suppression of angiogenesis [18,19,20].

CD4+ helper T-cells are further divided into five subsets, among which regulatory T-cells (Tregs) are most associated with the tumor microenvironment through the suppression of inflammatory responses [21]. The capacity of Tregs to suppress CD8+ cells may play a significant role in the immune evasion of CS tumors, as accumulation of Tregs has been linked to both CD8+ cell exhaustion and immune resistance. However, tumor cells have also been shown to reprogram Tregs through the secretion of CCL22 and CCL28 [22,23]. The tumor-induced reprogramming of Tregs has been implicated in the generation of a tumor-promoting TME through the suppression of tumor-adverse immune cell populations [24]. In this way, Treg-mediated immune response suppression has been widely studied in the TME, but further research is necessary to determine their specific role in CS.

Like other immune cell populations, T-cells remain under-investigated in CS. Nevertheless, due to their importance in immunotherapy, it is paramount to understand the presence and activation of T-cell populations in CS to adequately stratify patients for therapy. Research has identified T-cells within the CS microenvironment. Within a cohort of 61 CS samples, CD4+ cells were found in 21% of tumors. In a cohort of 62 CS samples, CD8+ T cells were identified in 44% of tumors. Further analyses revealed elevation in CD8+ levels in DD CS, with these T-cells present in 21% of CCS samples versus 90% in DD CS samples. Elevated CD8+ levels were also observed in high-grade (3) tumors as opposed to low-grade (1) [25]. These observations suggest a potential association between CD8+ T-cell infiltration and higher CS tumor grade.

Despite more rigorous CD4+ and CD8+ investigation in relation to the CS TME, less is known about non-conventional T-cell populations. Cell types such as γδ T cells and mucosal-associated invariant T-cells have been identified, but their role remains unspecified [23,25]. Further studies involving cell profiling will allow for further identification of non-conventional T-cell subtypes and, downstream, help to determine their function within the CS TME.

## 5. B-Cells

B-cells, another cell type differentiated from CLPs, are an integral part of the adaptive immune system and provide immunity against a variety of microorganisms, fungi, and parasites. In addition to producing antibodies, B-cells present antigens and secrete cytokines, thereby supporting T-cell responses [26].

Reports of the presence and function of B-cells within the chondrosarcoma TME are sparse. In 2021, a study conducted by Li et al. revealed the presence of small numbers of B-cells within the samples [27]. The identification of B-cells within the CS TME has also been approached using the surface protein CD20 as a marker. From IHC analysis, B-cells comprised 6.7 ± 3.8% of the DD CS TME, with statistically significant correlations between B-cell infiltration and stage of disease [28]. Furthermore, de Jong et al. reported a positive correlation between Bcl-2 expression and CS histological grade. Targeting of Bcl-xl using ABT-737, a small molecule BH3 mimetic inhibiting anti-apoptotic proteins in the BCL-2 family, significantly decreased tumor growth in a Swarm rat chondrosarcoma model [29]. This finding does not establish a functional role for B-cell infiltration, as BCL-2 family proteins are not specific to B cells, but targeting of B-cells may provide a new avenue for CS treatment. Although B-cell infiltration has been detected, the functional role of B-cells and their potential therapeutic targets remain unclear. Dose-limiting toxicity remains a concern, even with newer, more specific BCL-2 inhibitors like venetoclax.

## 6. Natural Killer (NK) Cells

Natural killer (NK) cells are lymphoid cells derived from CLPs. Depending on specific transcription factor expression, CLPs may differentiate into T cells, B cells, or innate lymphoid cells (ILCs) [30]. Researchers cite NKs as an ILC subtype, with many distinctions existing between the parent NK and ILC [31]. In terms of location, NKs are found within the bloodstream, while mature and differentiated ILCs are positioned within the tissue [32]. NK cells are cytotoxic in nature, allowing them to directly attack infected and cancerous cells.

Limited insights into the relationship between sarcoma and NK cells exist, with even less available information on CS and NK cells. Additionally, the results have been inconsistent between studies, further highlighting the need for further investigation and larger sample sizes.

A study by Sayitoglu et al. using an array of sarcoma samples, including CS, found low levels of NK cell infiltration. However, the elevated expression of NK receptors was associated with worse prognosis in this population [33]. Rademancher et al. investigated the impact of IL-12 overexpression, an activator of NK cells, on CS tumor growth in vivo using humanized mouse models. IL-12 cytokine overexpression significantly reduced tumor growth in the models, suggesting that IL-12 expression is key in initiating anti-tumor NK cell function. However, this study observed significant treatment-limiting side effects [34]. This observation was further supported by a study by Sayitoglu et al. showing that genetically modified GM NK-92 cells expressing DNAM-1 and NKG2D displayed higher affinity towards sarcoma tissues than their wild-type counterparts [33].

## 7. Neutrophils

Neutrophils are the most abundant type of leukocyte and the most abundant cell type within the body. These white blood cells originate in the bone marrow, where they differentiate to the neutrophil lineage from myeloid precursors [35].

Neutrophils play a compelling role in malignancies, where they can act as tumor suppressors and activators depending on neutrophil activation. When the enzyme neutrophil elastase is secreted, communication with histone H1 is initiated, triggering apoptosis in tumor cells. This mechanism is specific to malignant cells, allowing for healthy cells to survive and proliferate [36]. The neutrophils may then modulate the function of the cells to which they were signaled. ILC3, a type of innate lymphoid cell, closely related to natural killer cells, secretes chemokine CXCL8 and cytokine GM-CSF, which attract the neutrophils to a local environment and promote their survival, respectively. These signals ultimately contribute to the persistence of neutrophils and defense against tumorous cells [37].

Conversely, neutrophils may be manipulated and act as tumor progenitors. Tumor cells may recruit neutrophils to enhance their function in the same way that other immune cells can. In a study focusing on gastric cancer, Wang et al. found that tumor cells secreted GM-CSF, the same cytokine that recruits neutrophils in ILC3s. Once recruited, neutrophils upregulated PD-L1, which worked to suppress immune T-cells rather than cancer cells [38].

Neutrophil extracellular traps (NETs) are web-like structures made up of neutrophil DNA and are formed upon neutrophil death. A NET functions to entrap bacteria and other harmful cells. Research from McGill University provides evidence to suggest that tumor cells promote inflammation to increase NETosis and NET formation so that more tumor cells may become trapped in new locations, promoting metastasis [39]. Both tumor cell recruitment of neutrophils and NET influence on macrophages demonstrate how neutrophils may be hijacked. This makes the consideration of neutrophils difficult when considering cancers, as they may be simultaneously promoting the suppression and proliferation of tumor cells.

Although neutrophils have been observed within the CS microenvironment, few studies have investigated their impact on CS progression and outcome. In a retrospective study of 23 CS patients, Liu et al. observed an association between an elevated peripheral blood neutrophil-to-lymphocyte ratio (NLR) and patient survival. Patients displaying higher neutrophil levels (greater NLRs) faced worse overall survival prognoses than those who had lower neutrophil levels. In this study, NLR should be interpreted as a systemic inflammatory biomarker [40]. The correlation between high NLR infiltration and outcome was also observed in a case study involving a 72-year-old male patient with DD CS. The patient was characterized as having leukocytosis, with the greatest level of elevation in neutrophils. Elevated levels of G-CSF suggested a potential role for tumor-associated neutrophil recruitment, although this observation does not establish a causal relationship [41].

A retrospective study by Clara-Altamarino et al. yielded similar conclusions. From the cohort of 81 diagnosed patients over 18, 63.8% were classified as having high (>2.0) NLR. Furthermore, a high NLR was again associated with worse prognosis, supporting the potential prognostic value of NLR level as a non-invasive predictor [42]. Further studies incorporating both peripheral blood inflammatory markers and tumor-associated neutrophils are needed to determine whether systemic inflammation corresponds with the CS TME.

## 8. Immune Checkpoints

Immune checkpoints are regulatory molecules that help in controlling immune cell activity, especially in protection against overactivation. One of the most well-characterized immune checkpoint pathways is the PD-1/PD-L1 axis. Programmed cell death ligand 1 (PD-L1) acts as the associated ligand for programmed cell death protein 1 (PD-1). When PD-L1 binds to PD-1, an inhibitory signal is sent to the T-cells, reducing lymphocyte activation and subsequent proliferation to prevent excessive tissue damage during immune responses [43]. However, research has demonstrated that, in the generalized TME, this pathway may be exploited to suppress antitumoral T-cell responses, allowing for tumor cell survival [44,45]. This phenomenon has been observed in the CS TME. In an analysis of both the tumor and associated tumor-infiltrating lymphocytes (TILs) of 15 CS patients, PD-1 was identified in 33% of DD CS tumors and 53% of TILs, effectively demonstrating the presence of the associated immune checkpoint within the CS TME. Furthermore, positive PD-1 staining was found to have a statistically significant, positive correlation with a decreased time to metastasis [38]. This finding suggests that checkpoint-mediated immune suppression may contribute to the TME and tumor progression in aggressive CS subtypes. Clinically, the association between PD-1 expression and earlier metastatic progression raises the possibility that immune checkpoint expression may help identify patients with more aggressive disease, although this has not been validated as a predictive biomarker. Similarly, in a study evaluating 137 CS patients of varying subtypes, Kostine et al. demonstrated that PD-L1 expression was limited to DD CS samples. Furthermore, 41% of DD CS samples displayed a significant correlation between PD-L1 expression and elevated levels of T-cell infiltration. Although CS has generally demonstrated limited responsiveness to immunotherapy, this study suggests that certain CS subsets may possess an immune microenvironment potentially suitable for PD-L1 targeting [46]. However, PD-L1 expression alone does not establish responsiveness to PD-1/PD-L1 inhibitors. Prospective studies incorporating PD-L1 expression, tumor subtype, and clinical response are needed to determine whether these features can be used to identify patients likely to benefit from immunotherapy.

Another immune checkpoint protein relevant to discussions of T-cell regulation is B7-H3, also referred to as CD276. B7-H3 is a member of the B7 protein family, which has been shown to inhibit human T-cells. However, the immune pathway for B7-H3 is currently unclear, as its receptor is non-specific and its role as a costimulatory factor is debated [47]. Despite its ambiguity, B7-H3 expression has been identified within the TME. Revisiting research from Nota et al., an analysis of 48 C CS samples and 24 DD CS samples. B7-H3 expression was positive in 38% of C CS samples and 88% of DD CS samples, demonstrating higher expression in the more aggressive subtype. Insufficient information exists to draw concrete conclusions regarding the role of B7-H3 and its role in the CS TME. However, its higher expression in DD CS suggests that B7-H3 should be investigated as a potential marker of an immunosuppressive tumor phenotype. Functional studies and clinical investigations are needed to determine if this has a therapeutic relevance in CS [38].

## 9. Prospective Biomarkers

Biomarkers are biological components within the body—typically genes, proteins, or particular cells—that provide information regarding the presence, behavior, and progression of a disease. A small variety of biomarkers have been studied in relation to CS. Survivin is a protein that inhibits apoptosis (IAP). Per Jong et al., survivin was found to be overexpressed in 207 chondrosarcoma samples, with particular upregulation in the nucleus. This resistance to apoptosis could explain the proliferation of tumorous cells [48]. S100 protein, identifiable by its role in calcium-binding, was found to be expressed in 75% of chondrosarcoma samples in a study run by Karamchandani et al. However, there were only four samples, limiting statistical significance. Additionally, S100 was also identified in other sarcoma subtypes, including Ewing sarcoma, extraskeletal myxoid sarcoma, and rhabdomyosarcoma [49]. The presence of S100 in each subtype paired with a low statistical significance indicates that insufficient data is available to classify the protein as a biomarker.

Even less is known about biomarkers that can be linked to the CS TME, but there are many under investigation. A key family of proteins studied for their association with the CS TME is the vascular endothelial growth factor (VEGF) family. VEGF proteins are responsible for triggering angiogenesis, which is the process governing the formation of new blood vessels from previously existing ones [50]. A foundational study from Furumatsu et al. was one of the first to observe and provide evidence for the ability of CS to secrete VEGF, suggesting that it is a primary player in CS tumor angiogenesis [51].

Further investigations have since been undertaken and have demonstrated VEGF interacting with various proteins within the tumor and CS TME. In one study, chick embryos were injected with two different CS cell lines. The first cell line, JJ012/CCL5, contained increased levels of the proinflammatory cytokine CCL5. The second cell line, JJ012/CCL5-shRNA, was the opposite, being a CS cell line where CCL5 was knocked down. Implant assay results revealed the conditioned medium (CM) from the JJ012/CCL5 cell line with increased CCL5 increased the formation and growth of microvessels in comparison to the JJ012/CCL5-shRNA cell line. This same trend was observed when implemented in a nude-mouse model, providing evidence for CCL5 as an angiogenesis promoter when in cooperation with VEGF [52].

A similar experimental method was utilized in studying other regulators of angiogenesis. A study run by Lee et al. focused on adiponectin. Adiponectin is a protein involved in the regulation of glucose and insulin sensitization. Similarly, two cell lines were injected in both chick and mouse models, with the first cell line (JJ012/contro-shRNA) acting as a control and the second cell line (adiponectin-shRNA) having knocked down adiponectin. Decreased angiogenesis was observed in the CM from the knocked-down adiponectin–shRNA cell line [53]. A different study from Wang et al. examined the role of amphiregulin (AR), an epidermal growth factor receptor ligand. Results showed that AR promoted both the production of VEGF-A and subsequent angiogenesis through the AK/c-Src/PKCδ pathway [54]. Resistin, a cysteine-rich hormone associated with inflammation and diabetes, has also been identified as an angiogenesis promoter by Chen et al. [55]. These three studies provide evidence for the classification of various proteins as angiogenesis promoters when their function is considered in conjunction with VEGF. This positions VEGF as a central biomarker within the CS TME, with multiple upstream signaling molecules appearing to converge on VEGF to promote vascular remodeling and disease progression. However, these hypotheses have not been studied enough, and further research should be conducted to confirm their roles.

Methods of treatment related to angiogenesis-associated biomarkers are sparse but exist. The study about CCL5 and VEGF from Liu et al. not only identifies key players within angiogenesis but highlights a potential intervention to slow or even prevent tumor proliferation. This can be seen using an additional tool, miR-199a. This was also utilized in both the chick and mouse models and, when injected, blocked the effects of CCL5 on angiogenesis [52]. Another method of treatment was proposed by Sun et al., focusing on CXCR4. CXCR4 is a chemokine receptor that regulates cell migration and, through activation of the CXCR4/SDF-1 axis, can increase the secretion of VEGF and downstream CS angiogenesis. Using a mouse xenograft model, the CXCR4 antagonist AMD3100 was injected into some of the mice. Mice injected with AMD3100 experienced decreased VEGF levels, tumor growth, and metastases to the lung [56]. While both mouse models show promise, additional studies must be performed to validate their efficacy.

Biomarkers associated with hypoxia have also been linked to CS. Hypoxia occurs when oxygen demand outweighs oxygen supply, and responses can vary between different tissues. Hypoxia-associated biomarkers such as hypoxia-inducible factor 1-alpha (HIF-1ɑ) may act as an additional level of insight into the CS TME, as hypoxia has been demonstrated to increase tumor angiogenesis and adaptation [57]. Research from Lin et al. verifies the expression of HIF-1ɑ in a CS cell line after being subjected to low oxygen conditions for at least 30 min. Furthermore, a positive correlation was found between HIF-1ɑ and VEGF expression, suggesting a relationship between elevated HIF-1ɑ and increased tumor angiogenesis [58]. Further studies from Sun et al. demonstrated that increased HIF-1ɑ expression under hypoxic conditions was correlated with an increase in CXCR4 expression in JJ cells. Interaction between CXCR4 and its ligand along the CXCR4/SDF-1 axis activates ERK signaling and MMP1 expression, which subsequently results in the degradation of the extracellular matrix. Degradation of the extracellular matrix, in turn, has been linked to increase CS migration and invasion, suggesting that HIF-1ɑ may play a role in both the promotion of angiogenesis and act as a driver of invasion [56].

Prospective biomarkers also come from the antigen-presenting class (HLA) of proteins. Antigen-presenting molecules, as the nomenclature suggests, are those involved in the processing and display of innate and foreign antigens. This presentation allows for immune cells, particularly CD8+ cells, to both identify and respond to the antigen [59]. The identification of HLA biomarkers in the CS TME would be significant, as they could indicate whether the tumorous cells are able to present antigens to T-cells and, subsequently, if immune cells would be able to respond and attack these tumor cells. Research has demonstrated HLA expression in CS samples. In an analysis of 50 C CS samples and 20 DD CS samples, HLA-A expression was positive in 8% of the C CS samples and 85% of DD CS samples. HLA-A was also found to be elevated in higher grade C CS samples, with a mean expression of 39 ± 23 for the samples of grade 2–3 and 16% ± 13 for grade 1 samples. The same trend was observed in HLA-B expression, with mean expressions of 49% ± 25 for samples of grade 2–3 and 27% ± 2 for grade 1 samples [23]. Considered together, this evidence suggests that HLA expression may be indicative of both predictions of CS subtype and aggression. However, not enough research has been done to confirm these HLA molecules as biomarkers.

Immune cell populations in chondrosarcoma have been studied by a few authors for their prognostic and diagnostic roles. Exosomes play a role in enhancing tumor-promoting macrophages in the setting of hypoxia, resulting in metastases and increased angiogenesis [60]. However, the correlation of CD68-positive CD163-positive tumor-associated macrophages with metastasis and prognosis has been inconsistent [4]. Chondrosarcoma tumor grade is associated with immune cell populations, as well-described by Li et al. In this study, 98 chondrosarcoma cases were analyzed by mass cytometry (CyTOF), WES and flow cytometry (22 cases). Three tumor immune microenvironment states were identified: myeloid derived stem cell (MDSC) dominant, associated with high grade and myxoid tumors; immune exhausted, correlated with high grade tumors; and immune desert, associated with lower grade chondrosarcomas. Immunotherapy may be effective for the immune-exhausted group [27]. PDL1 expression is documented predominantly in grade 3 and dedifferentiated chondrosarcomas, although a correlation with prognosis is not identified [33,61].

Overall, insufficient data exist to suggest that each of these biomarkers can be definitively linked to the CS TME or even to CS as a broader classification or are of clinical utility. This signifies that further investigation should be undertaken to gain greater knowledge on predictive factors of chondrosarcoma.

## 10. Modern Approaches for Study of the Tumor Immune Microenvironment

To better understand the TME and its role in CS, it is important to be familiar with different techniques that may be employed in its study. Single-cell RNA sequencing (scRNA-seq) is used to identify the gene expression of various cells, allowing for types to be identified within the TME [12,18]. CyTOF and high-dimensional flow cytometry can also be used for cell-type identification using surface and intracellular proteins as markers [27]. Three-dimensional tumor co-culture models can also be used to study the CS TME, providing a method for investigating interactions in a physiologically relevant environment [17]. Functional perturbation and depletion methods alter or remove specific immune-cell populations, signaling pathways, or genes to determine their causal effects on tumor growth and immune activity. Studies of this variety allow for examination of the impacts of particular TME components.

## 11. Discussion

The CS tumor immune microenvironment is far more heterogeneous and clinically relevant than previously appreciated, yet remains poorly defined. While macrophages, T-cells, B-cells, immune checkpoints, NK cells, neutrophils, and various biomarkers have been implicated in phenotypic changes of CS tumors across different studies, most existing data is limited to small cohorts and retrospective analyses. As a result, our current understanding of how individual immune cell populations influence CS progression, prognosis, and therapeutic response—particularly across C CS and DD CS subtypes—remains incomplete.

However, emerging evidence proposing the existence of CS patient subsets responsive to immune-based strategies is encouraging but inconclusive. As the current literature stands, it is difficult to pinpoint the most effective potential biomarkers and immunotherapy treatments. Performing additional studies to further develop these methods is essential for taking steps towards less invasive CS treatments. Before meaningful clinical advances can occur, there is a critical need for larger, prospective studies. It is important to acknowledge the difficulty of this necessity, however. With CS being such a rare disease, it can be difficult to obtain statistically significant sample sizes, particularly in a clinical setting. In this way, another important research goal for the future is prioritizing the creation of a cross-institutional network of CS samples and data so that more robust research efforts can be undertaken.

While the current findings underscore the biological importance of the CS immune landscape, they primarily serve to emphasize how much remains unknown. This review enhances CS research efforts through the aggregation and organization of existing evidence. While immune targets have yet to become more specified and limited studies on potential immunotherapy and clinical options exist, a deliberate, coordinated research effort will be necessary to translate these preliminary insights into effective, clinically meaningful therapies. Of specific importance are the development of robust research models and coordinated efforts for the collection of high-quality human specimens for translational and clinical research.

## 12. Conclusions

The CS TME is increasingly recognized as an important contributor to tumor progression, immune evasion, and therapeutic resistance, although it remains incompletely characterized. The current evidence demonstrates heterogeneity in immune cell composition across chondrosarcoma subtypes and tumor grades, with tumor-associated macrophages, T-cells, B-cells, natural killer cells, neutrophils, and immune checkpoint pathways contributing to the complex tumor microenvironment. However, much of the available evidence is derived from small cohorts, observational studies, or experimental models, and the functional significance of many immune cell populations remains uncertain. Even though immune classifications regarding certain cell expressions suggest that patients have distinct immune profiles relevant to immunotherapy, immune markers and immune cell populations have not been sufficiently validated for diagnostic, staging, or treatment purposes. Future studies should prioritize larger prospective cohorts and standardized immune-profiling methods to better define immune phenotypes. Such efforts may identify reliable biomarkers and therapeutic targets and improve the development of personalized strategies for patients with advanced chondrosarcoma.

## Data Availability

All data are available upon request.

## Abbreviations

Chondrosarcoma (CS), conventional CS (C CS), dedifferentiated chondrosarcoma (DD CS), tumor immune microenvironment (TIME), classically activated macrophages (M1), alternatively activated macrophages (M2), lipopolysaccharide (LPS), interferon gamma (IFN-γ), tumor necrosis factor (TNF-α), tumor-associated macrophages (TAMs), colony-stimulating factor 1 receptor macrophage (CSF-1R+), common lymphoid progenitors (CLPs), cytotoxic T-cells (CD8+), helper T-cells (CD4+), regulatory T-cells (Tregs), programmed cell death ligand 1 (PD-L1), programmed cell death protein 1 (PD-1), tumor-infiltrating lymphocytes (TILs), natural killer (NK), innate lymphoid cells (ILCs), neutrophil extracellular traps (NETs), neutrophil/lymphocyte infiltration (NLR), inhibitor of apoptosis (IAP), vascular endothelial growth factor (VEGF), conditioned medium (CM), amphiregulin (AR), hypoxia-inducible factor 1-alpha (HIF-1ɑ), antigen-presenting class (HLA), mass cytometry (CyTOF), single-cell RNA sequencing (scRNA-seq).
